# Mapping Organelle Motion Reveals a Vesicular Conveyor Belt Spatially Replenishing Secretory Vesicles in Stimulated Chromaffin Cells

**DOI:** 10.1371/journal.pone.0087242

**Published:** 2014-01-29

**Authors:** Guillaume Maucort, Ravikiran Kasula, Andreas Papadopulos, Timo A. Nieminen, Halina Rubinsztein-Dunlop, Frederic A. Meunier

**Affiliations:** 1 Queensland Brain Institute, Clem Jones Centre for Ageing Dementia Research, The University of Queensland, Brisbane, Queensland, Australia; 2 School of Mathematics and Physics, The University of Queensland, Brisbane, Queensland, Australia; UPR 3212 CNRS -Université de Strasbourg, France

## Abstract

How neurosecretory cells spatially adjust their secretory vesicle pools to replenish those that have fused and released their hormonal content is currently unknown. Here we designed a novel set of image analyses to map the probability of tracked organelles undergoing a specific type of movement (free, caged or directed). We then applied our analysis to time-lapse z-stack confocal imaging of secretory vesicles from bovine Chromaffin cells to map the global changes in vesicle motion and directionality occurring upon secretagogue stimulation. We report a defined region abutting the cortical actin network that actively transports secretory vesicles and is dissipated by actin and microtubule depolymerizing drugs. The directionality of this “conveyor belt” towards the cell surface is activated by stimulation. Actin and microtubule networks therefore cooperatively probe the microenvironment to transport secretory vesicles to the periphery, providing a mechanism whereby cells globally adjust their vesicle pools in response to secretagogue stimulation.

## Introduction

Transport of intracellular organelles by molecular motors to their target destination is critical to power polarity and cellular processes such as endocytic trafficking, secretory pathways and axonal retrograde transport in neurons [1]. How the microenvironment conveys organelles to their target destination at steady state and how this transport is affected by signaling is currently unknown. We have designed a novel set of image analyses that uses tracked organelle trajectories to map their probability of undergoing specific type of movements (free, caged and directed) relative to their position in the cell. Mapping organelles motion has the potential to reveal regions of the cell that transport or capture organelles by precisely quantifying the probability of single organelles to undergo free, caged or directed motion. More importantly, such analysis could shed new lights into how activation of a given signalling process can globally affect such functional regions.

To test our analysis we use labelled secretory vesicles from neurosecretory cells. In these cells, hormones and neuropeptides are stored in secretory vesicles formed at the level of the *trans*-Golgi network. The actin and microtubule networks control the transport of secretory vesicles [2]–[5] to the plasma membrane and their release by exocytic fusion in response to stimulation. Whilst neurons replenish their synaptic vesicles through local endocytosis and recycling [6], very little is known of how neurosecretory cells spatially replenish their secretory vesicles [7]. Unlike neurons, these cells do not locally recycle secretory vesicles. Even though after fusion the vesicular membrane and core are recovered, there is no evidence to suggest that they are locally reused [8], [9]. Most studies point to a classical secretory pathway involving packaging in the *trans* Golgi network and maturation, [10] followed by docking, priming and exocytic fusion. We therefore hypothesize that some steps in this secretory pathway are controlled by secretagogue stimulation allowing vesicles to spatially adjust their vesicle pools to replenish those that have undergone fusion.

We used time-lapse z-stack confocal imaging of secretory vesicles from transfected bovine chromaffin cells to map the global changes in vesicle motion and directionality occurring upon secretagogue stimulation. Here, we report the active recruitment of secretory vesicles towards the plasma membrane in response to stimulation. We found that vesicles undergoing free, caged or directed motion were spatially segregated and differentially affected by secretagogue stimulation. A defined region abutting the cortical actin network appeared to actively transport secretory vesicles towards the cell surface, we tested actin and microtubule depolymerizing drugs and found that they dissipated this vesicular “conveyor belt”. Therefore both cytoskeleton networks cooperatively probe the microenvironment to recruit and transport free moving secretory vesicles from the centre to the periphery of neurosecretory cells to replenish the pools of secretory vesicles lost during stimulation.

## Results and Discussion

**Figure 1:**
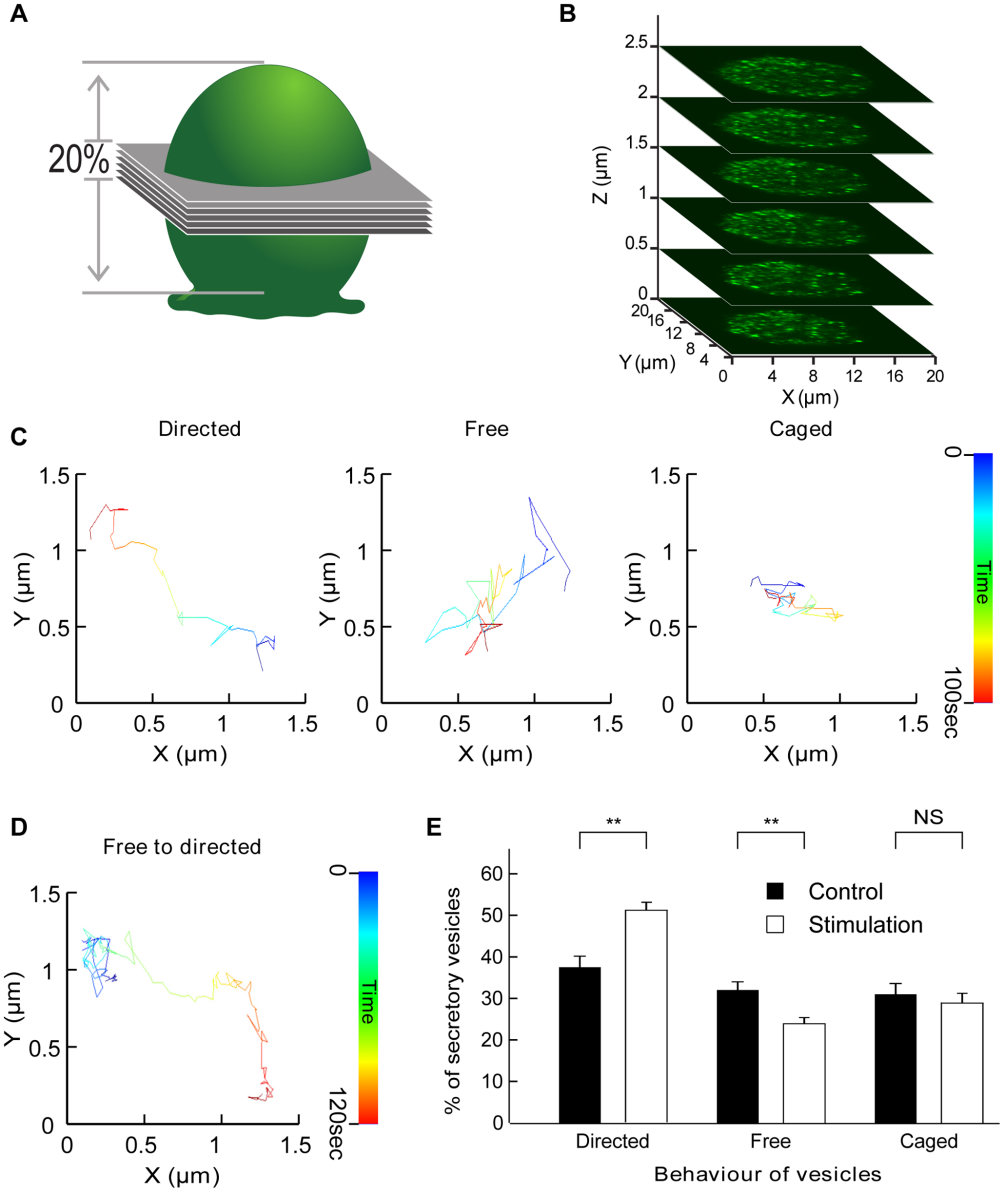
Acquisition and tracking of secretory vesicles in chromaffin cells and categorization of their motion. Chromaffin cells expressing hGH-GFP were imaged by confocal microscopy. (**A**) Six optical slices were acquired to obtain a 3 µm z-stack encompassing 20% of the total height of a chromaffin cell (**B**). (**C**) Time-coded prototypical trajectories of vesicles tracked for 100 sec illustrating the three different types of vesicular movement as indicated. (**D**) Time-coded prototypical vesicle trajectory showing a switch from caged behaviour (blue) to directed motion. (**E**) Comparison of percentages of vesicles in each of the three different motion pools in control conditions and during nicotine (10 µM) stimulation (N = 7 cells, n = 1159 tracked vesicles). Note the significant increase in directed motion and the parallel decrease in the percentage of vesicles undergoing free diffusion. **p<0.01 (paired *t*-test), NS: not significant.

Time series of the z-stack from chromaffin cells expressing GFP-tagged human growth hormone (hGH-GFP) were carried out to monitor and analyse the change in secretory granule (SG) behaviour taking place upon secretagogue stimulation. To determine where within the cell the switch from free to directed motion occurs upon stimulation, we monitored the distance from each tracked vesicle to the closest plasma membrane. As chromaffin cells are round and the z-stack (centred in the middle of the cell) encompassed approximately 20% of the total cellular volume, the closest plasma membrane was located in the x–y plane (Figure 1A–B). To minimize potential errors, we restricted our analysis to regions located within 5 µm of the edges of the cell. The centre of the cells was not considered because of uncertainties regarding the closest membrane direction. Fitting parameters allowed us to sort vesicles according to their type of movement (Figure S1A). Three types of movements (caged, free or directed) are present in unstimulated chromaffin cells (Figure 1C) and switches in movement behaviour were detected in response to secretagogue stimulation (Figure 1D). The percentages of vesicles undergoing caged, free or directed movement was extracted (1431 vesicles tracked from 8 cells) before (control) and immediately after nicotine treatment (stimulation). A significant increase in the percentage of SGs undergoing directed motion was observed in parallel with a decrease in the number of free vesicles (Figure 1E). These results suggest that a significant number of vesicles are switching from free to directed diffusion during stimulation, consistent with the selective recruitment and directed transport of vesicles. Previous studies using evanescence microscopy, which restricts the analysis of vesicle motion to a limited penetration depth, have not detected such a switch in vesicular diffusion mode [11], [12]. This suggests that the switch from free to directed movement could occur deeper within the cell, a hypothesis consistent with the recruitment and transport of SGs towards the plasma membrane to replenish the pool of vesicles that has undergone fusion. Furthermore, previous studies have pointed to a reduction in the number of caged vesicles upon stimulation as they undergo fusion with the plasma membrane [13]. Our result shows that the percentage of caged vesicles is unchanged by stimulation, suggesting that the pool of caged vesicles undergoing exocytosis is actively replenished following stimulation.

**Figure 2:**
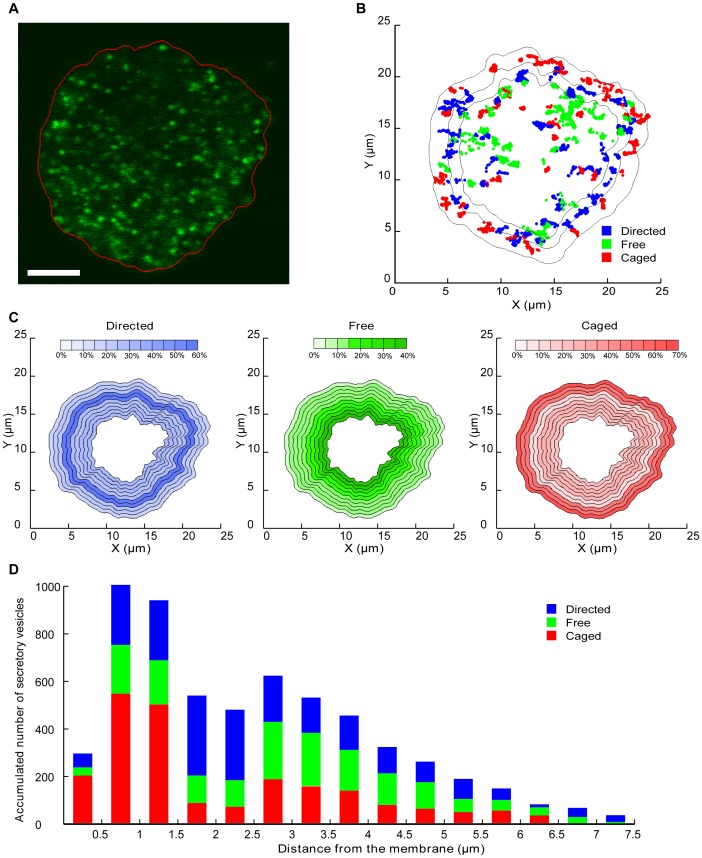
Mapping of secretory vesicle behaviour and trajectories relative to the plasma membrane. (**A**) Example of a single confocal image of a chromaffin cell expressing hGH-GFP with an edge detection algorithm applied to every frame of the 3D movies to retrieve membrane position at all times. (**B**) Example of the map of the same cell generated after nicotine (10 µM) stimulation (only 3 min of acquisition is displayed) with colour-coded vesicle trajectories. The external black line represents the average detection of the plasma membrane for this cell and the internal lines denote the edges of the 0–1.5 µm and 1.5–2.5 µm zones. (**C**) Data from 7 stimulated cells (n = 724 vesicles) were used to generate maps of the average percentage of vesicles in a given motion state, based on their distance to the plasma membrane. Note that the chosen cell shape used for representation is prototypical. For analysis purposes, the cells have been divided into 0.5 µm zones. The central part of the cell is not represented due to insufficient data and uncertainties regarding the closest membrane. (**D**) Histogram of accumulated vesicle positions depending on their motion and distance to the membrane.

We next performed edge detection (see methods) on every frame to monitor the external membrane position over the acquisition time (Figure 2A). We then mapped the vesicles’ trajectories and colour-coded their motion type on the plot of the average membrane position (Figure 2B). As expected, most of the caged movements were restricted to the region close to the plasma membrane as previously established[13]–[16]. Interestingly, however, vesicles undergoing directed motion also seemed to lie within the subcortical region of the cell (Figure 2B), with the centre of the cell being predominantly filled with free vesicles. We then analysed the percentage of vesicles displaying each of the three types of motion depending on their distance from the nearest plasma membrane (Figure 2C–2D). The results were displayed on a prototypical cell shape (Figure 2C). As suggested above, the subcortical region located between 1.5 and 2.5 µm from the plasma membrane mostly contained vesicles undergoing directed motion (60%; Figure 2C). This region was also relatively underpopulated, as most directed vesicles tended to remain a shorter time in this location. We detected a gradual decrease from the centre (40%) to the periphery (5%) of the cell in the percentage of vesicles undergoing free diffusion. The region directly adjacent to the membrane was clearly underpopulated, containing mostly caged vesicles (Figure 2D), in good agreement with the role of the cortical actin network as a diffusion barrier [5].

**Figure 3:**
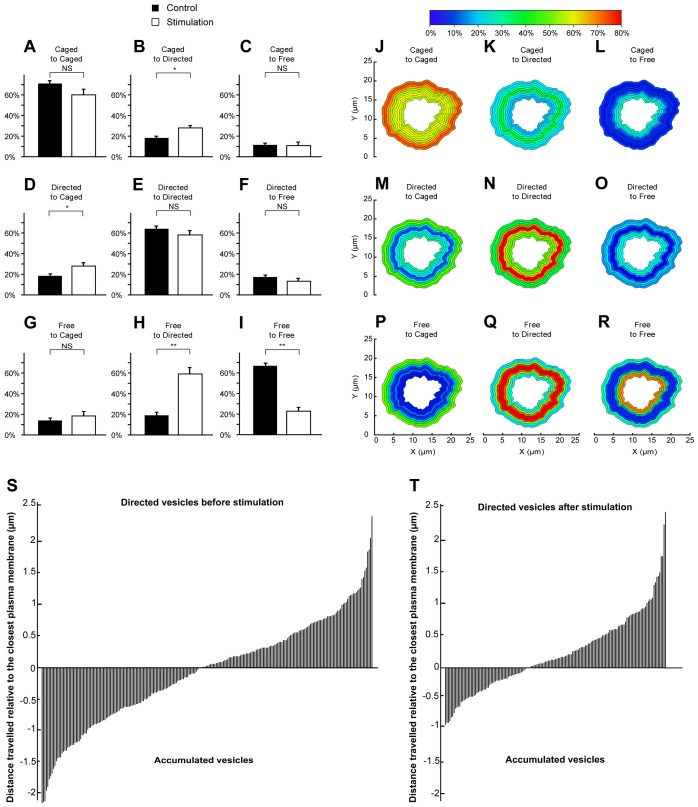
Quantification and mapping of single vesicle motion and directionality switches following stimulation. Secretory vesicles tracked before and during secretagogue (Nicotine, 10 µM) stimulation were monitored and the type of motion mapped and compared. The percentages of caged vesicles (**A**, **B** and **C**), directed vesicles (**D**, **E** and **F**) and free vesicles (**G**, **H** and **I**) switching to caged (**A**, **D** and **G**), directed (**B**, **E** and **H**) or free (**C**, **F** and **I**) motion was calculated and compared in control and stimulated conditions. Observed percentages of switches in vesicle motion were mapped relative to the distance of the vesicle from the plasma membrane (**J–R**). The directionality of directed vesicles was established relative to the closest plasma membrane. Negative values represent vesicles travelling away from the membrane, whereas positive values denote those moving towards the plasma membrane. Null values represent vesicles that are directed transversally to the membrane. (**S**) Directed vesicles before stimulation (N = 7 cells, n = 234 vesicles). (**T**) Directed vesicles after stimulation (N = 7 cells, n = 157 vesicles).

These results strongly suggest that vesicles can change their behaviour in response to stimulation, depending on their location in the cell. To determine the precise nature of these switches, we compared the changes in vesicle motion occurring spontaneously (in resting conditions) with those taking place during stimulation. The spontaneous and activity-driven motion changes were then analysed and sorted into 9 pools. In resting conditions, the majority of the caged vesicles remained caged (Figure 3A), with only a few switching to directed (Figure 3B) or free movement (Figure 3C). This trend remained largely unchanged in response to stimulation (Figure 3A–C). We detected a small albeit significant increase in the percentage of vesicles switching from caged to directed motion (Figure 3B). This was surprising considering that the overall percentage of caged vesicles did not change between control and stimulated conditions (Figure 1E). However, an equally significant increase in the percentage of vesicles switching from directed to caged motion was detected (Figure 3D). This is consistent with an active replenishment of the caged vesicles that have undergone fusion. At rest, the majority of directed vesicles remained in the same mode (Figure 3E), with only a small percentage of vesicles switching to the free mode (Figure 3F). These trends were unaffected by stimulation of exocytosis (Figure 3E–F). Most of the free vesicles also remained free at rest, with only a few switching to caged or directed movement (Figure 3G–I). However, upon stimulation, a large percentage of free vesicles (83±5%) switched to directed motion (Figure 3H).

To examine where these switches were taking place, we calculated for each 0.5 µm layers the percentages of switches due to stimulation from originally caged behaviour vesicles to caged behaviour (Figure 3J), free behaviour (Figure 3K) or directed behaviour (Figure 3L). The same study was done for originally free vesicles (Figure 3M–O) and originally directed vesicles (Figure 3P–R). As expected, most of the originally caged vesicles remained caged near the plasma membrane (Figure 3J). The small number of caged vesicles switching to directed motion lay within 1.5–3 µm of the plasma membrane (Figure 3K). This suggests that some of the directed vesicles found in the subcortical zone (Figure 2C) also originated from previously caged vesicles. Caged vesicles switching to the free mode occurred deeper within the cell (Figure 3L), consistent with a slow uncaging of these vesicles. Interestingly, vesicles undergoing directed motion mostly switched to caged movement in the vicinity of the plasma membrane (Figure 3M), although the occurrence of this particular switch in this zone was low as a high percentage of directed vesicles remained in the directed mode in this region (Figure 3N). Vesicles changing from directed to free movement occurred deeper within the cell (Figure 3O). At the periphery, the movement of the small population of free vesicles had a high chance of becoming restricted (Figure 3P). Importantly, the vast majority of freely moving vesicles lying in the zone 1.5–3.5 µm from the plasma membrane became directed (Figure 3Q). This demonstrates that an activity-dependent recruitment of originally free secretory vesicles takes place within this catchment area for active transport. We also noted that the instant speed of directed vesicles in the subcortical conveyor zone was significantly increased by secretagogue stimulation (Table 1). The remaining free-moving vesicles had a higher chance of being found deep within the cell (Figure 3R). Taken together, our data indicate that the activity-dependent recruitment of free and, to some extent, caged vesicles is elicited in a specific area of chromaffin cells in response to secretagogue stimulation. Considering the gradual change in the percentage of vesicles found to be free, from higher in the centre of the cell to lower towards the periphery, we predicted that the global movement of directed vesicles was likely to translocate vesicles between these regions. We established the directionality of the directed vesicles located in the subcortical zone by analyzing the direction taken by the tracked vesicles relative to the closest plasma membrane. The difference in distance to the plasma membrane between the start and the end of the track was measured. At rest, directionality was evenly distributed between movement towards or away from the plasma membrane (Figure 3S). However, the neutral directionality of directed vesicles significantly changed following stimulation (Figure 3T). Indeed, the ratio between vesicles moving towards the membrane and those travelling in the opposite direction increased from 1∶1 to 2∶1, implying the active recruitment and translocation of vesicles towards the plasma membrane in response to stimulation. The vesicles moving away from the membrane were not able to travel as far as in resting conditions, which might result from cytoskeletal rearrangements favoring active recruitment and vesicular transport towards the membrane.

**Table 1:** Change in secretory vesicle instant speed in the subcortical conveyor and the cortical exchange zones.

		Caged		Free		Directed	
Type of motion	Stimulation	Instant speed(µm/s, ×10^−2^)	n	Instant speed(µm/s, ×10^−2^)	n	Instant speed(µm/s, ×10^−2^)	n
Subcortical conveyor zone	Before	3.90±0.12	700	4.33±0.08	1347	4.18±0.10	887
	During	3.70±0.01^NS^	628	4.14±0.09^NS^	1123	5.00±0.15***	578
Cortical exchange zone	Before	3.89±0.08	971	4.31±0.06	2082	4.31±0.07	1944
	During	3.30±0.06***	1577	3.91±0.07***	2043	4.37±0.09^NS^	1172

For all the tracked vesicles, instant speed at all tracking points of the vesicle was recorded with Imaris and sorted depending on the cellular location of the vesicle and its type of motion (subcortical transfer zone is 1.5–2.5 µm from the membrane, and cortical exchange zone is 0–1.5 µm from the membrane). The average instant speed of the vesicles in each given state was then calculated in the two areas. Note the significant change in instant speed of directed vesicles in both areas of the cell. Mean ± S.E.M.

* p<0.05,

*** p<0.0001, ^NS^: not significant.

**Figure 4:**
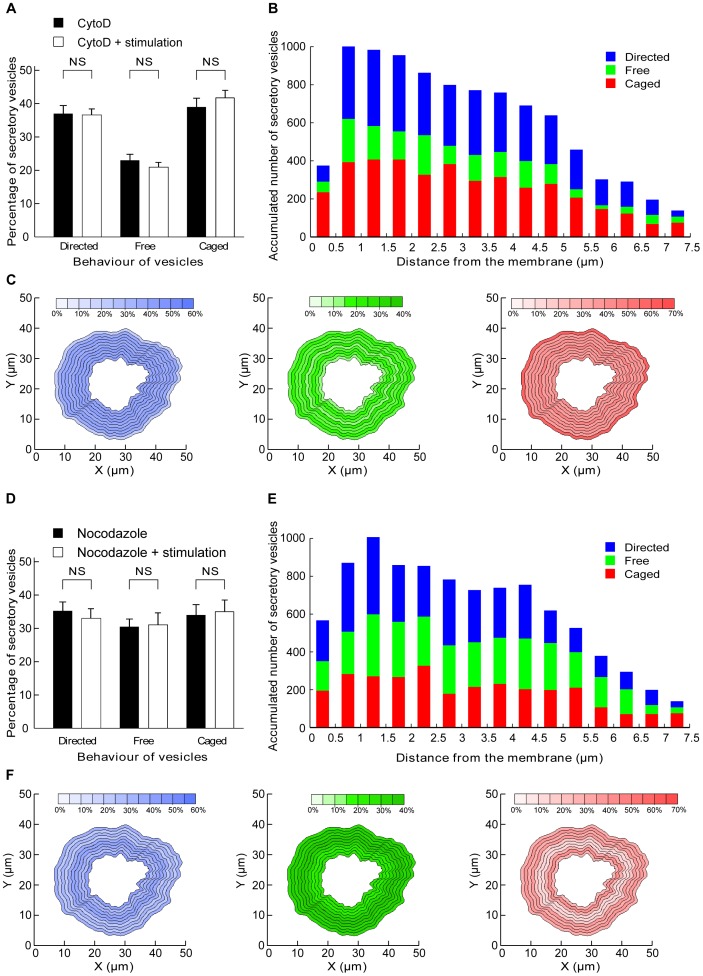
Blocking actin and microtubule polymerization interferes with the replenishment mechanism. (**A, B, C**) Chromaffin cells were incubated with cytochalasin D (10 µM) for 20 minutes before imaging and stimulation. (**A**) Comparison of percentages of vesicles lying in the three different pools in control conditions and during nicotine stimulation (10 µM) (N = 5 cells, n = 978 vesicles). (**B**) Histogram of accumulated vesicle positions depending on their motion and distance from the membrane. (**C**) Data from stimulated cells were used to generate maps of the average percentage of vesicles in a given motion state, relative to their distance from the plasma membrane. For analysis purposes, the cells have been divided in 0.5 µm zones. The central part of cells is not represented due to insufficient data and uncertainties regarding the closest membrane direction. (**D**, **E**, **F**) Chromaffin cells were incubated with nocodazole (20 µM) for 20 minutes before imaging and nicotine stimulation. (**D**) Comparison of percentage of vesicles lying in the three different pools in control conditions and during stimulation of exocytosis following nicotine (10 µM) stimulation (N = 4 cells, n = 629 vesicles). (**E**) Histogram of accumulated vesicle positions depending on their motion and distance from the membrane. (**F**) Data from stimulated cells were used to generate maps of the average percentage of vesicles in a given motion state, based on their distance from the plasma membrane.

To test this hypothesis, similar analyses were performed using inhibitors of actin polymerization (cytochalasin D [17] and latrunculin [13], [18]). hGH-GFP-expressing chromaffin cells were treated with cytochalasin D (10 µM for 20 min) and imaged by confocal microscopy before and during nicotine stimulation as in Figure 1. Analyses of the percentage of vesicles undergoing free, directed or caged movement in resting cells (Figure 4A) revealed similar results to those found in untreated cells (Figure 1E). However, stimulation did not significantly change any of the motion types (Figure 4A), in sharp contrast to the change detected in stimulated untreated cells (Figure 1E). The free vesicles had therefore lost the ability to be actively recruited and transported, pointing to a critical function of actin polymerization in generating microenvironmental forces conveying vesicles to the plasma membrane. As a result, the different pools of vesicles were more randomly distributed within the cells (Figure 4B–C). Moreover, the freely diffusing vesicles were present throughout the cell (Figure 4C), further confirming the dissipation of the gradual change in free motion detected in untreated cells (Figure 2C). Noticeably, the region abutting the plasma membrane did not exhibit a high density of caged vesicles, with these vesicles now being spread out throughout the cell (Figure 4C). However, the region close to the plasma membrane remained low in vesicles, suggesting that the cytochalasin-resistant cortical actin network can still act as a diffusion barrier. Similar results were obtained following pre-incubation with latrunculin (Figure S2).

Similar to the effects of latrunculin and cytochalasin D treatment, nocodazole-treated cells displayed no detectable change in vesicle behaviour in response to nicotine stimulation (Figure 4D–F). This suggests that both actin and microtubule networks underpin the recruitment and directed transport of secretory vesicles towards the plasma membrane in an activity-dependent manner. Our global analysis of single vesicle movement revealed three major zones: a central zone, containing mainly free vesicles, a subcortical zone in which vesicles are transported towards the plasma membrane in an activity-dependent manner, and a peripheral zone containing mostly confined vesicles. As a result, the number of free vesicles gradually decreases from the centre to the periphery of the cell where they are captured by the cortical actin network before undergoing fusion. Interfering with the actin and tubulin cytoskeleton networks dissipated this gradient. These findings reveal a novel role for the actin and microtubule cytoskeletons in which they actively probe the microenvironment to couple the overall transport of vesicles to stimulation, thereby ensuring the replenishment of the secretory vesicles undergoing fusion. More generally, our image analysis can be applied to other types of organelles to investigate the mechanism regulating their global movements within the cell.

## Materials and Methods

### Reagents and Drugs

Nicotine, cytochalasin D, latrunculin A and nocodazole were purchased from Sigma-Aldrich (Australia).

### Cell Preparation

Adrenal chromaffin cells were extracted from bovine adrenal medulla, cultured [19] and transfected with pXGH5 vector-encoding hGH [20] for 48 hours before use. Cells were bathed in Buffer A (145 mM NaCl, 5 mM KCl, 1.2 mM Na_2_HPO_4_, 20 mM Hepes-NaOH, 2 mM CaCl_2_, pH 7.4) containing 5.6 mM D-glucose, before addition of nicotine.

When cytoskeletal inhibitors were used (cytochalasin D (2 µM) or nocodazole (20 µM)) these were pre-incubated with the cells for 20 minutes prior to imaging.

### Confocal Microscopy

Chromaffin cells were then washed 3 times with buffer A and examined using a Zeiss 510-Meta confocal microscope using a x63 oil-immersion objective (0.75 NA). A region of interest containing the studied cell was determined and a time-lapse z-stack acquisition was performed. The thickness of the 3D image was 2.5 µm and a stack was acquired every 2 seconds. Stimulation was achieved by addition of nicotine (10 µM) after 3 to 4 minutes of imaging, with the cell being imaged for a further 3 to 4 minutes following stimulation. Images were examined with Zen software (Oberkochen, Germany).

#### Tracking

A compromise between acquisition rate, image resolution and number of slices acquired was required to retrieve sufficient information to perform post-processing analysis (0.33 Hz image acquisition, 512×512 images, 6×0.5 µm optical slices). The z-stack centred on the equator of the cell only encompassed 20% of the cell height. From the images extracted with Zen (Figure S1B), we reconstructed a 3D movie using Imaris software (Bitplane, Zurich, Switzerland). We realized that taking the z position into account was an important source of error due to the limited thickness of our images. In view of the relative roundness of chromaffin cells, we assumed that movements in the x–y plan were similar to those in the x–z and y–z plans, and only took into account x–y movements. Mean square displacement was calculated and fitted using homemade Matlab programs. Vesicles tracked for fewer than 30 frames (1 min) were excluded from the analysis.

Filters were applied to enhance contrast between the fluorophores and the cell background. We then tracked the vesicles with the autoregressive algorithm in Imaris (Figure S1C). The algorithm was set up to track 0.5 µm objects, which could be traced for more than 30 frames (1 minute) without disappearing (no frame jump). Due to the crowded nature of the cells, a compromise between the number of vesicles tracked and the quality of tracks was made. The x,y,z coordinates of every tracked vesicle were then extracted and exported as an Excel worksheet. Even though the tracking was performed in 3 dimensions, the subsequent analysis was performed only through the x and x axes given that the limited thickness of our acquisition introduced large uncertainties on the z-axis and considering that the spherical shape of chromaffin cells allowed us to assume that the movements in the x–z or y–z directions were similar to those in the x–y plane.

### Data Analysis

#### Mean square displacement

From the coordinates over time, we calculated the mean squared displacement (MSD) [21] for each vesicle using:
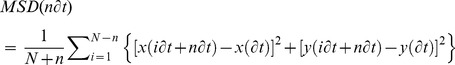
(1)with N being the number of tracking points of the vesicle, 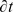 the time between two points, and 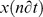 and 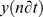 the coordinates of the point at the time 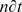.

The MSD of particles tracked before and after stimulation was calculated for both parts of the trajectory in order to study changes in behaviour. For these vesicles, a minimum of 20 points before and after stimulation was required in order to get significant MSD calculations. These calculations were performed using self-developed Matlab programs (Mathwork).

#### Sorting

By fitting the MSD curves with the appropriate equations, we identified different types of motion. A good fit to a straight line is indicative of random diffusion, also called Brownian motion
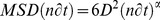
(2)with a coefficient *α* = 1, D is the diffusion coefficient defined as 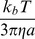 where *k_b_* is the Boltzmann constant, *T* is the temperature, *η* is the viscosity of the medium and *a* is the diameter of the particle.

In directed diffusion a drift velocity is added to the equation
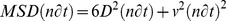
(3)

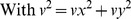



The anomalous sub-diffusion model considers the presence of energy traps, a caged diffusion is characterized by the MSD’s negative curvature in the plots. The vesicle motion is then confined in cage-like tether.
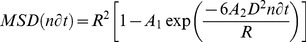
(4)where 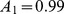 and 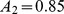 (fixed constants for spherical cage) and 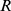 is the radius of the spherical cage in which the particle is free to diffuse with the diffusion coefficient 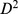.

Fitting and sorting were conducted using self-developed Matlab programs.

#### Edge detection

The position of the membrane was determined for each cell. Acquisition was performed in the middle of spherical cells over a 2.5 µm thick layer. To approximate the membrane position, we projected the 3D image on the z-axis and applied filters to reject the background and blur the image in order to get a smooth image. This was possible due to the large amount of fluorescence located at the periphery of the cell. A self-coded edge detection program using Labview (National Instruments) was applied on each frame of our movie to get the edge position at each time for our cell. We then extracted the points defining the membrane position at each time point.

#### Distance to the closest membrane

We used a Matlab self-coded program to determine for each time the closest membrane point and its distance to the vesicles at each time point. For each vesicle we then obtained a full set of information including its position, behaviour, and distance to the closest plasma membrane for each timepoint.

## Supporting Information

Figure S1 **(A)** Example of a 3D image obtained with the confocal acquisition. **(B)** Example of the tracks obtained from a z-stack. Note the difference between the number of tracks retained and the number of fluorescent vesicles seen in the previous image. **(C)** Examples of the 3 different types of mean square displacement (MSD) fitting curves from the vesicles’ trajectories (x–y plane).
(TIF)

Figure S2 Chromaffin cells were incubated with latrunculin (10 µM) for 20 minutes before imaging and stimulation. (**A**) Comparison of percentages of vesicles lying in the three different pools in control conditions and during nicotine (10 µM) stimulation (N = 3 cells, n = 596 vesicles). (**B**) Example of the map generated after nicotine stimulation (3 min acquisition) with colour-coded vesicle trajectories. The external black line represents the average detection of the plasma membrane for this cell and the internal lines denote the edges of the 0–1.5 µm and 1.5–2.5 µm zones. (**C**) Data from stimulated cells were used to generate maps of the average percentage of vesicles in a given motion state, relative to their distance from the plasma membrane. (**D**) Histogram of accumulated vesicle positions based on their motion and distance from the membrane.
(TIF)
