# Supplementary figures and images for: Mapping Organelle Motion Reveals a Vesicular Conveyor Belt Spatially Replenishing Secretory Vesicles in Stimulated Chromaffin Cells
Source: PLoS One. 2014 Jan 29;9(1):e87242. doi: 10.1371/journal.pone.0087242 (PMC3906151; doi:10.1371/journal.pone.0087242)

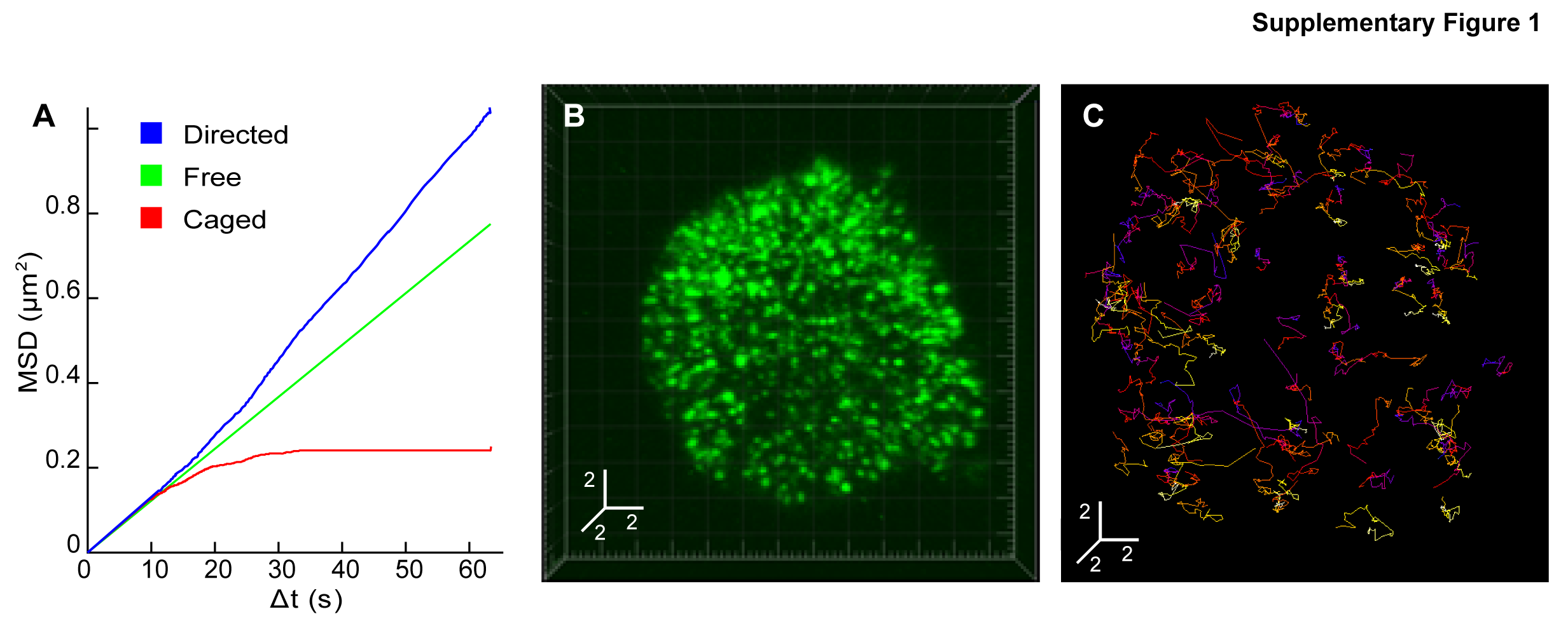

Supplement: Figure S1 — (A) Example of a 3D image obtained with the confocal acquisition. (B) Example of the tracks obtained from a z-stack. Note the difference between the number of tracks retained and the number of fluorescent vesicles seen in the previous image. (C) Examples of the 3 different types of mean square displacement (MSD) fitting curves from the vesicles’ trajectories (x–y plane). (TIF) [file pone.0087242.s001.tif]

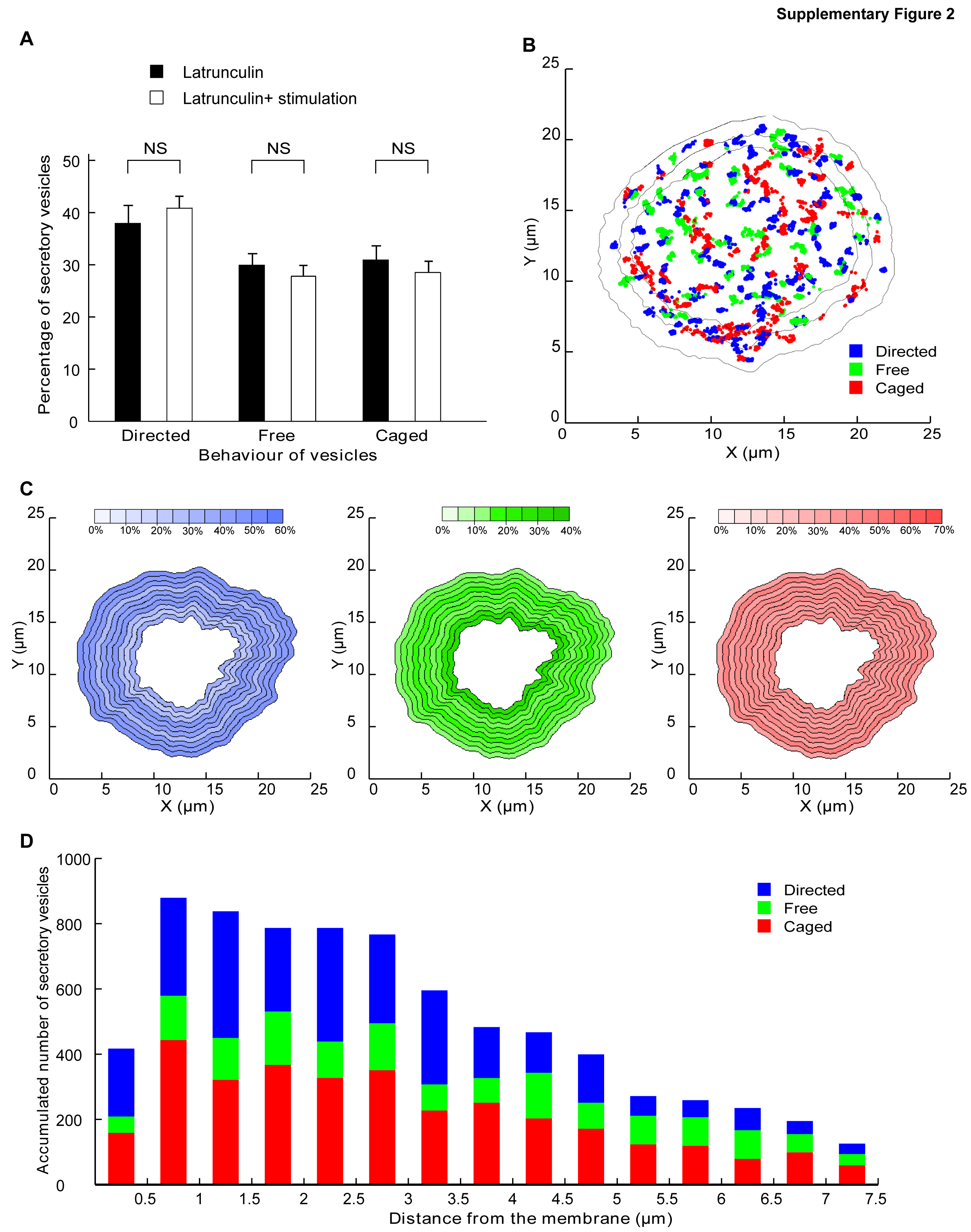

Supplement: Figure S2 — Chromaffin cells were incubated with latrunculin (10 µM) for 20 minutes before imaging and stimulation. (A) Comparison of percentages of vesicles lying in the three different pools in control conditions and during nicotine (10 µM) stimulation (N = 3 cells, n = 596 vesicles). (B) Example of the map generated after nicotine stimulation (3 min acquisition) with colour-coded vesicle trajectories. The external black line represents the average detection of the plasma membrane for this cell and the internal lines denote the edges of the 0–1.5 µm and 1.5–2.5 µm zones. (C) Data from stimulated cells were used to generate maps of the average percentage of vesicles in a given motion state, relative to their distance from the plasma membrane. (D) Histogram of accumulated vesicle positions based on their motion and distance from the membrane. (TIF) [file pone.0087242.s002.tif]
